# Grasping and Releasing Agarose micro Beads in Water Drops

**DOI:** 10.3390/mi10070436

**Published:** 2019-06-30

**Authors:** Federica Vurchio, Pietro Ursi, Alessio Buzzin, Andrea Veroli, Andrea Scorza, Matteo Verotti, Salvatore Andrea Sciuto, Nicola Pio Belfiore

**Affiliations:** 1 Department of Engineering, University of Roma Tre, via della Vasca Navale 79, 00146 Rome, Italy; 2 Department of General Surgery and Surgical Specialties “Paride Stefanini”, Sapienza University of Rome, Viale del Policlinico 155, 00161 Rome, Italy; 3 Department of Information Engineering, Electronic and Telecommunications, Sapienza University of Rome, Via Eudossiana, 18, 00184 Roma, Italy; 4 Department of Mechanical, Energy, Management and Transportation Engineering University of Genoa, 16145 Genoa, Italy

**Keywords:** microgrippers, agarose, MEMS, comb-drives, microscopy, characterization, minimally invasive surgery, micromanipulation

## Abstract

The micromanipulation of micro objects is nowadays the focus of several investigations, specially in biomedical applications. Therefore, some manipulation tasks are required to be in aqueous environment and become more challenging because they depend upon observation and actuation methods that are compatible with MEMS Technology based micromanipulators. This paper describes how three grasping-releasing based tasks have been successfully applied to agarose micro beads whose average size is about 60 μm: (i) the extraction of a single micro bead from a water drop; (ii) the insertion of a single micro bead into the drop; (iii) the grasping of a single micro bead inside the drop. The success of the performed tasks rely on the use of a microgripper previously designed, fabricated, and tested.

## 1. Introduction

The development of the ability of manipulating micro objects has a high impact on several applications, such as biomedical operations and minimal invasive surgery, as only representative examples. About a hundred different microgrippers have been presented in literature [1,2] for a variety of applications, but there are still several issues which attracted researchers’ attention.

In 2002 a microgripper has been built by combining silicon fingertips fabricated by a micro-machining process and conventional piezoelectric actuators [3]. This microsystem was able to move three micro-objects of diameter about 80μm from one position to another.

A superelastic alloy microgripper has been fabricated by electro-discharge machining and integrated with electromagnetic actuators and piezoelectric force sensors [4]. For the analyzed microgripper, the superelastic alloy (NiTi) presented larger displacement capability than stainless steel.

Cell biomechanics has been investigated by an integrated platform that combines both force sensing and actuation [5]. To achieve this goal a submersible lateral force sensor has been used. Another example of water proof microsystem is a three-axis MEMS based accelerometers presented in 2012 [6].

MEMS Technology based microgrippers have been used to grasp and release micro- and nano-particles [7,8] or HeLa cells [9] inside a fluid channel. These micro objects have been suspended in water and aligned by using ultrasonic fields. The adopted microgrippers provided 100μm opening between fingers.

A 3D structure, electroplated nickel fabricated, magnetically actuated micro-assembled microrobot prototype, with 1 mm overall size, has been used to test a multi-axis MEMS force and torque sensor [10]. Recently, a more complex structure has been also investigated, consisting of a micropositioning stage and a set of microgrippers fabricated from a single silicon-on-insulator wafer [11].

In 1995 micro Physics has been applied to micromanipulation tasks and many interesting phenomena involved in the handling of micro objects were identified. Attractive forces have been modeled and experimental tests have been carried out [12]. These results have been used to develop a new micro manipulation strategy.

Interactive forces between micro objects and a microgripper surface have been studied also in 1998 [13]. In particular, van der Waals’s, surface tension and electrostatic forces have been studied in air and some differences between the micro and macro worlds have been exemplified for grasping, opening and releasing (or adhere). The surface roughness of the end-effector surface has been identified as a possible means to reduce van der Waals’s forces.

The above mentioned progress suggested possible developments in surgery [14]. Microrobotic techniques have been also used, for example, for closing abnormal communication between the atria of the heart during a beating-heart catheter-based procedure [15]. In fact, MEMS based devices are promising to improve minimally invasive laparoscopic or endoluminal operations: for example, laparoscopic sleeve gastrectomy (LSG) with cruroplasty [16], surgical treatment of gastrointestinal stromal tumors of the duodenum [17] and colovesical fistula surgery [18]; or Endoluminal loco-regional resection by TEM (Transanal Endoscopic Microsurgery) [19,20,21], and Low Rectal Anterior Resection (LAR) [22].

More examples of applications of MEMS to the development of biomedical instruments are available in literature [23,24,25]. For example, the study of the mechanical characteristics of cells gives information about their functionality and their state of health [26]. For this reason, cells viscoelasticity [27,28], the mechanical response of muscle cells subjected to elongation [29], the stiffness skeletal muscle cells [30], and the mechanical properties of tissue-engineered vascular constructs [31] have been investigated. The mechanical characterization of human red blood cells has been also approached by means of electrothermal MEMS microgrippers [32,33,34]. Microactuation has been also considered in viscous dielectric media [35,36,37] or underwater [38,39]. To this aim microgrippers should be tested in operational conditions and environments that is a very challenging task not fully investigated in literature.

In the present investigation agarose has been used to test the gripper capabilities. Agarose-based polymers are naturally inert and biocompatible materials whose mechanical properties are similar to those of soft tissues. They are also very suitable to be employed as carriers for various biomedical applications [40]. Furthermore, they are often used as porous matrix/scaffold for biotechnological applications, from tissue engineering to cell immobilization and wastewater filtering [41].

Agarose-based micro beads are a well-established helping tool in the biomedical field for a variety of applications such as protein detection, DNA hybridization and in general as a mean to immobilize biomolecules in order to support reactions and to permit a faster molecular detection with higher sensitivity and lower reagent consumption, making it an ideal choice for microscaled lab-on-chip devices [42]. More specifically, microbead-based microfluidic platforms provide a boost in the planned chemical or biochemical reactions, increasing the degree of interaction between the biomolecules and the functionalized surfaces. As an example, such set-ups are able to substantially improve the hybridization efficiency of DNA strands in order to obtain a more sensitive detection of various biomarkers for several health disorders. Moreover, an enhancement can be observed in food contaminant detection when pre-functionalized agarose micro-beads are employed in these kind of operating systems [43]. Agarose micro beads are suitable as sensors for a wide range of microscaled immunoassays, representing a good physical support for optimized antibody capturing and antigen-antibody interactions, due to an intrinsic nanoporous structure and high surface-to-volume ratios [44]. Furthermore, microfluidic systems with the goal of performing single-cell DNA-extraction and DNA-purification recently include an encapsulation and incubation phase of single cells in agarose beads or droplets in order to enhance the quality of the resulting genetic analysis and improve the observation of mutations circumstances as a key to study, undestand and properly treat cancers [45].

This paper describes an experimental campaign aiming at reporting three micromanipulation tasks that have been performed on micro beads having variable sizes, from 45 μm to 165 μm (wet).

The grasping-releasing basic operations have been tested in and outside a water drop and then the following tasks have been successfully documented: (i) the extraction of a single micro bead from a drop; (ii) the insertion of a single micro bead into the drop; (iii) the grasping of a single micro bead inside a water drop.

The experimental activities described in this paper much owe to the past years, during which a new microgripper has been developed by the team, from the early stage of concept design to the operational testing, through the following phases: design [46,47,48], optimization [49,50,51], simulation and control [52], fabrication [53,54], mechanical characterization [55], actuation design [56] and testing [57], and, finally, operational [58] and functional [59,60,61] testing. Although the results of the present investigation (see Section 3) refer to the manipulation of micro objects whose overall size spans from 20 μm to 165 μm, there are some efforts to develop fabrication methods [62] toward the extreme miniaturization of microgrippers that could grasp even objects downsized by about one more order of magnitude.

## 2. Materials and Methods

The described experiments consist of a combination of basic maneuvers, such as grasping, handling and releasing, applied by the developed microgripper to agarose micro beads in an aqueous solution.

### 2.1. The Agarose Microbeads

**Figure 1:**
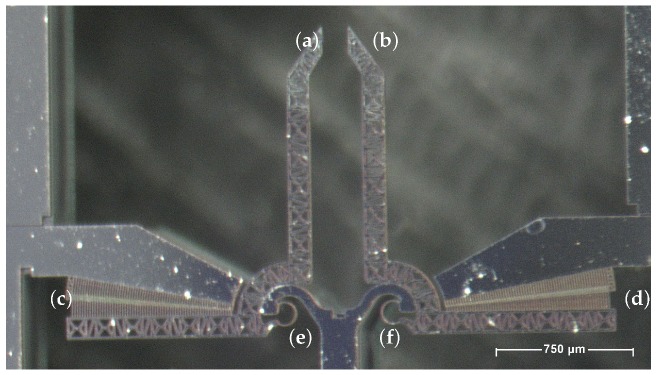
The Device Under Test (DUT): left (**a**) and right (**b**) jaws, left (**c**) and right (**d**) comb drives, and left (**e**) and right (**f**) CSFH hinges.

After an in-depth study, Sepharose© CL-4B agarose-based micro beads were chosen for the proposed experiments. The micro-beads have diameters which span from 45μm to 165μm in their wet state (at 23±1${}^{\circ }$C). In their dry state these beads may shrink down to 20μm. All the explained features and the affinity with the biomedical environment, together with the compatibility in terms of dimensions, make these micro beads a good candidate to test the manipulation capabilities of a micro-surgical apparatus in a hypothetical operational scenario. For this purpose, a microgripper prototype, shown in Figure 1, has been employed.

### 2.2. The Device Under Test (DUT)

The device under test (DUT) is a lumped compliance MEMS-Technology based system embedding two compliant hinges, namely, two Conjugate Surface Compliance Hinge (CSFH), that have been positioned in order to allow the two jaws to rotate around a fixed revolute axis. The mobile axes are activated by means of rotary comb drives that use electrostatic principle of actuation. Deep Reactive Ion Etching (DRIE) allowed the microsystem to achieve reasonable aspect ratios [53] (e.g., 8, as the result of the device thickness 40μm by the elastic curved beam width 5μm). The microsystem has an overall size of 2000μm×1500μm and an operating grasping window of 150μm×150μm, which implies an operating grasping volume of 150μm×150μm×40μm.

### 2.3. The Experimental Setup

In the present experimental campaign, the microsystem has been operated in air, while only the jaws tips penetrated the water drop surface to manipulate the agarose beads in water. After the extraction of the beads from the drop, the microgripper jaws maintained the bead in air.

**Figure 2:**
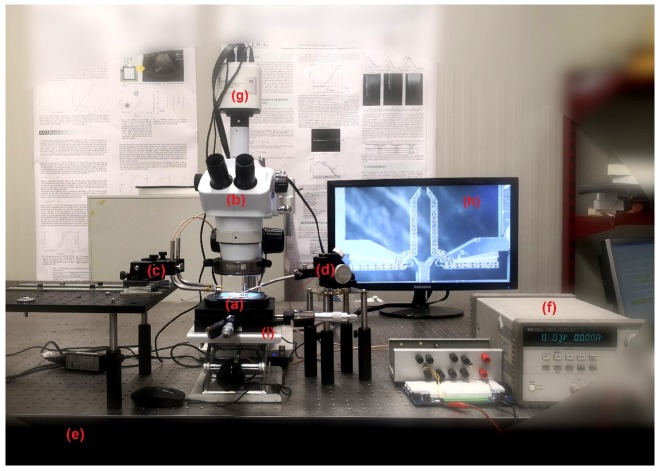
Experimental setup: microgripper (**a**), optical microscope (**b**), micropositioners with embedded probe arms and tungsten needles (**c**), micropositioner with spoon-shaped metal probe (**d**), pneumatic suspension table (**e**), supply voltage with protection circuit (**f**), embedded camera for images acquisition (**g**), monitor (**h**), and instrumented support with micrometric screws (**i**).

**Figure 3:**
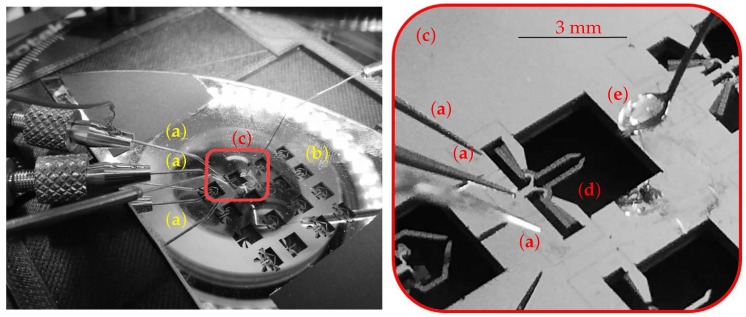
A detailed view of the the DUT and the probe arms: probes (**a**), wafer area with micro machined windows (**b**), operating area (**c**), DUT (**d**) and water drop (**e**).

In order to correctly carry out the experiments, it was necessary to face many challenges, such as the DUT tips motion toward the drop, their insertion into the aqueous medium, the grasping operation and their extraction from the drop. All these tasks require synergistic efforts by a gripper actuation apparatus that has to be carefully coordinated with DUT and water drop handling system. A semiautomatic procedure has been applied to grasp the beads, basically consisting of two phase movement, where a gross motion is followed by a second phase of fine tuning and grasping. The latter action has been implemented by using two electrodes for independently applying the voltages to the two sets of mobile fingers of the comb drives and one to the ground plate. For this purpose, 3 tungsten probes were placed by using X/Y/Z high resolution micropositioners in order to bring the electrical potential to the electrodes from a power supply circuit and correctly control the actuation tasks. The approaching and removing procedures were carried out using a spoon-shaped metal probe, which was fixed to a micropositioner as well, with the aim of hosting a water drop on its tip. A Light Trinocular Microscope (LTM) embedding a digital camera was employed to monitor the experiment. An overall view of the entire experimental setup is represented in Figure 2 and Figure 3.

In Table 1, the overall components of the experimental setup are reported.

The identification of the actual values of design, operational and control parameters is a fundamental issue for the success of the target task. Some values have been already identified by measurements or numerical simulations, some of them are introduced in the present paper, while some other parameters will be studied next year. For the sake of completeness, Table 2 reports some important operational parameters for the microgripper.

In the next section, the main phases necessary for the correct execution of the experiment are described: the preparation of the Sepharose CL-4B agarose-based micro beads in aqueous solution, the positioning of the water drop near the microgripper jaws, the grasping and release phases and the bead grasping.

### 2.4. Sample Preparation

As a preliminary phase, the agarose micro beads underwent a coloring procedure by means of a blue food dye to be better distinguished and recognized from the aqueous solution. Blue food coloring E 132 was preferred more than other types of dyes in order not to alter the chemical structure and the features of the beads. The pre-colored beads were then diluted in deionized water. Consequently, droplets of the resulting solution were collected and placed on top of the spoon-shaped probe with the help of a syringe.

### 2.5. Gripper Preparation

Once the droplets have been positioned near the microgripper jaws, the grasping/releasing phases are performed by micro-positioning the device and then applying voltage by means of three tungsten probes electrically connected to the power supply circuit. The water drop containing the micro beads is sustained by a spoon-shaped metal probe that is guided by a micro-positioning guide.

## 3. Results

In this section the results of three different micromanipulation tasks are reported:

(i) the extraction of a single micro bead from a drop;
(ii) the insertion of a single micro bead into the drop;
(iii) the grasping of a single micro bead inside a water drop.

These three tasks have been performed by using a semiautomatic procedure that makes use of gross motion micro positioning probes to approach the drop with 3 degrees of freedom (DoF) motion and fine gripping motion provided by externally applied voltage.

The experiments have been carried out at a speed that was low enough to exclude dynamic effects. The pictures in the three series have been taken at variable time intervals, with an average rate of 1 picture every 6 s. An average of one successful grasping every 4 attempts has been experienced during the tests. This success frequency can be improved by modifying agarose beads size and concentration, drop size, water evaporation and adhesion.

### 3.1. Extraction of a Single Micro Bead from a Drop

**Figure 4:**
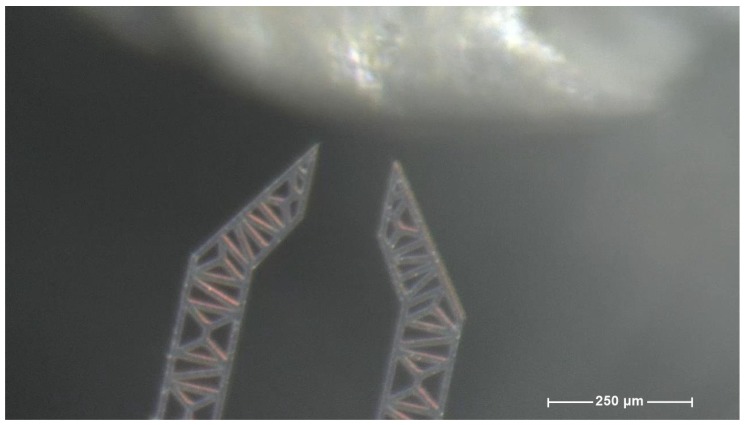
A frame from the sequence illustrated in the Figure 5 (grasping).

Using the equipment described in the previous paragraph some attempts have been made in order to extract one single agarose bead from a single drop. Figure 4 shows the operational field where the action takes place.

**Figure 5:**
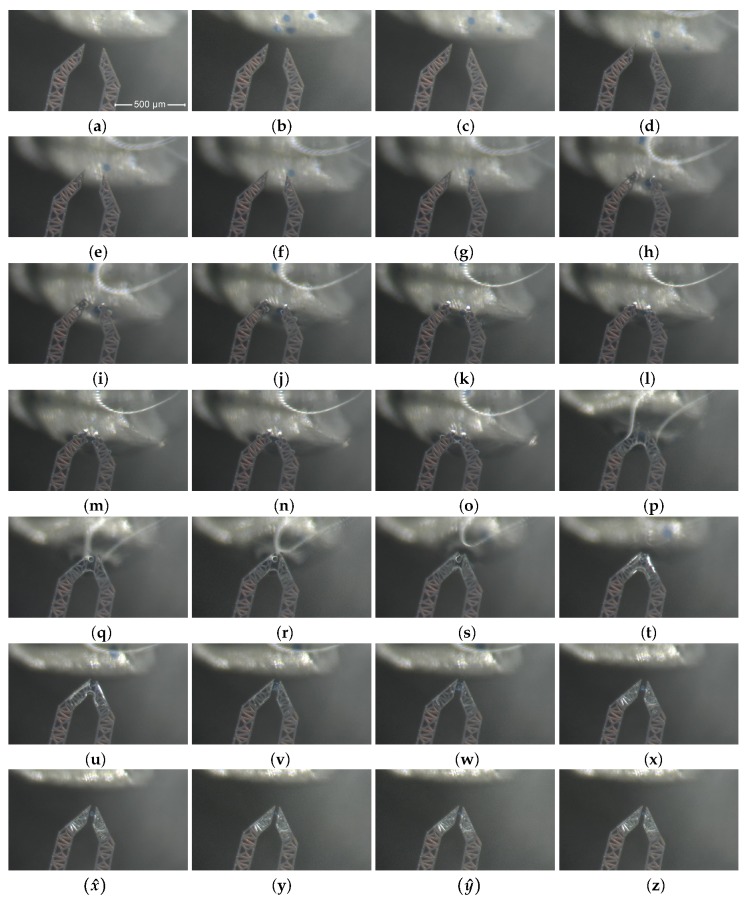
A complete grasping sequence: approaching, from (**a**) to (**f**); contact and insertion, from (**g**) to (**k**); grasping (**l**); exit from the drop, from (**m**) to (**p**); extraction, (**q**), (**r**) and (**s**); residual water evaporation from the jaws, from (**t**) to (**v**); bead progressive grasping and agarose shrinking, from (**w**) to (**z**).

Figure 5 presents through the frames from (**a**) to (**z**) a time ordered sequence of views of the operational field. The sequence is described by the following stages.

- First, the micro-gripper is set in its initial state (normally open, no voltage applied) and the water drop is guided toward the gripping device by operating the spoon-shaped droplet host micro-positioning device, as shown in Figure 5 from (**a**) to (**g**).
- The jaws-tips are then inserted into the water drop, as shown in Figures from (**h**) to (**k**).
- Once the tips are within the drop, a combination of micro-metric displacements of the tips and the drop allows one or more micro beads to enter inside the operating window area of the jaw-tips; usually, this operation require few minutes only, the waiting time mainly depending on the beads density (units per drop); the bead centering in the monitored pane is made via direct observation, while the transverse centering is done by using the microscope focusing.
- When a single micro bead is centered in the operating volume, the grasping phase is activated and the device is actuated by supplying a gradually increasing voltage from zero up to 24 V to the jaw-connected electrodes and keeping null the voltage on the ground electrode. This induces the gradual closure of the jaws and a successful bead grasping, as shown by frames from (**l**) to (**o**). The DUT is now in its grasping state.
- The water solution is then moved away from the closed grip system, operating the spoon-shaped droplet host micro-positioning device, as shown by frames from (**p**) to (**s**), until the bead is completely extracted, as in frame (**t**).
- During removal motion, part of the residual water on the jaw-tips evaporates, as illustrated in Figure 5 (**u**).
- Finally, a change in the bead dimension occurs due to evaporation phenomena. In fact, the passage from a wet to a dry environment implies a significant and progressive shrinking, as shown by frames from (**v**) to (**z**).

### 3.2. Insertion of a Single Micro Bead into the Drop

The second task (ii) consists in an operation which is opposite to the first task: the insertion of a single micro bead into the water drop. Since there is no other way to insert an agarose micro bead between the microgripper jaws, this task could take place only after the first task (i) had been completed. Task (ii) was performed not more than a minute later than task (i) had completed.

**Figure 6:**
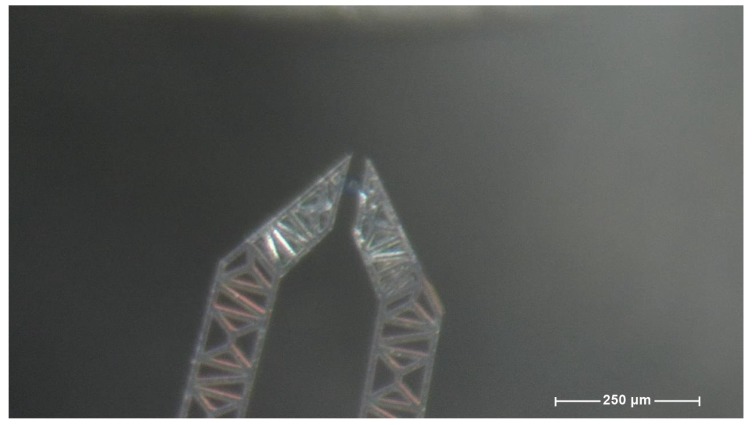
A frame from the sequence reported in the Figure 7 (releasing).

**Figure 7:**
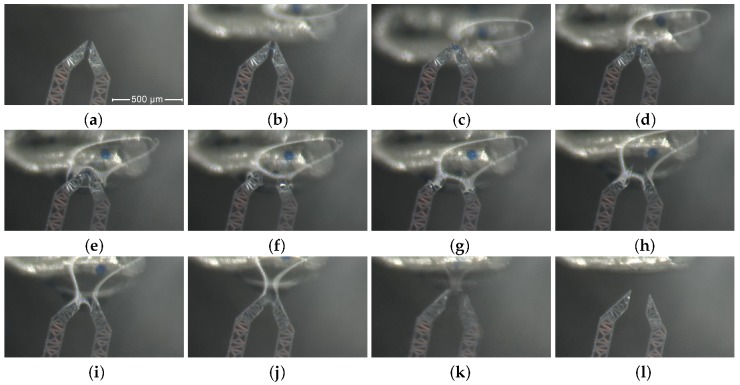
A complete releasing sequence: one bead hold by the jaws (**a**); progressive approaching, (**b**) and (**c**); contact with the drop and insertion (**d**) to (**e**); releasing (**f**); exit from the drop, from (**g**) to (**l**).

The operating window is documented by Figure 6. Figure 7 shows how task (ii) has been carried out, through the frames from (**a**) to (**l**).

- An agarose bead grasped by the device jaws is shown in Figure 7 (**a**).
- The water drop progressively approaches the device, as illustrated by frames (**b**) and (**c**).
- The micro bead is successfully inserted in the drop of solution, as reported by frames (**d**) and (**e**).
- The micro bead is then successfully released, as documented in Figure 7 by frames (**f**) and (**g**): in this phase, the voltage applied to the device is decreased down to 0 V.
- Finally, the device is extracted again from the water solution, as reported in frames from (**h**) to (**l**).

### 3.3. Grasping of a Single Micro Bead inside a Water Drop

An additional experiment has been considered as a third task (iii): the grasping of a micro bead inside a water drop. This particular task is crucial to perform new ways for dynamic characterization of cells and other types of microstructures, paving the way for new experimental micromanipulation scenarios. In fact, this particular device could be used in the future for the static force, stiffness, viscous and dynamic characterization of more complex cells or micro-structures.

**Figure 8:**
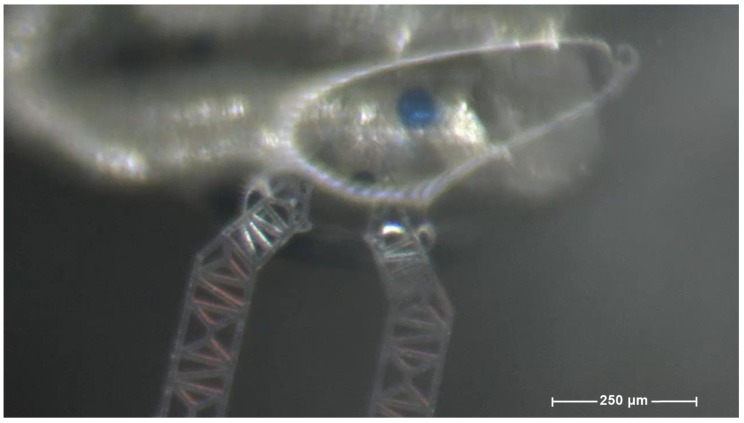
A frame from the sequence illustrated in the Figure 9 (grasping).

**Figure 9:**
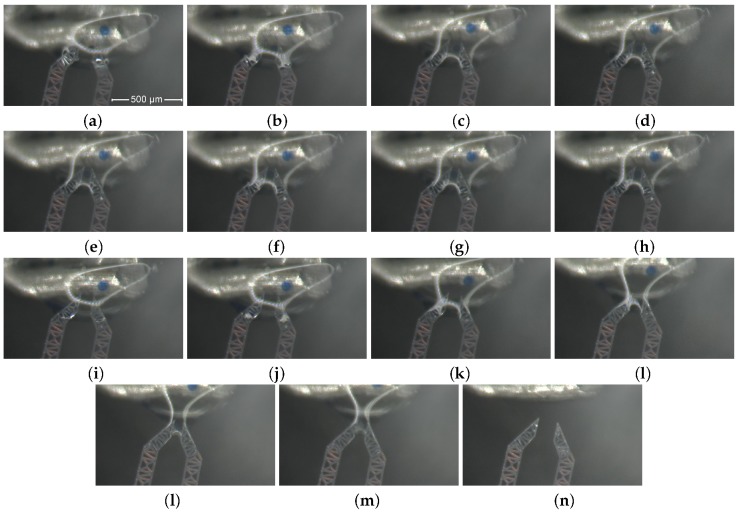
A sequence that illustrates the gripper jaws grasping a bead inside the water drop: progressive approaching to the agarose bead, from (**a**) to (**c**); contact with the bead(**d**); grasping, from (**e**) to (**h**); releasing (**i**); exit from the drop, from (**j**) to (**n**).

Figure 8 and the sequence of Figure 9, from (**a**) to (**n**), show the main steps of this experiment.

- First, the agarose bead is progressively approached, as depicted by Figures from (**a**) to (**c**).
- Then, the jaws grasp one agarose bead as illustrated in frame (**d**).
- Frames from (**e**) to (**h**) describe how the bead is squeezed.
- After that, the bead is released and let to go (**i**).
- The final sequence of frames, from (**j**) to (**n**), represents the extraction and removal of the jaw-tips from the drop.

## 4. Discussion

The experimental activity confirmed that many adhesion forces have a certain impact on gripping and releasing actions. In fact, due to scaling effects, adhesion forces such as van der Waals’s, electrostatic, surface tension, hydrogen bonding and liquid bridge force become more important than the inertial actions. These forces certainly make the micromanipulation of micro object rather difficult. For example, particles are difficult to be released because they can adhere to the surface of the gripper. Adhesion can be affected by various factors, such as humidity, temperature, surrounding medium, material and surface conditions. Arai et al. [12] showed that it is possible to reduce the adhesion force effects by controlling this kind of parameters: van der Waals force can be reduced by increasing the surface roughness; liquid bridge forces can be neglected in dry environment (humidity less than 9%); electrostatic forces can be also reduced by using conductive materials as thin film coatings. Furthermore, based on experimental measurements, Arai et al. [13] proposed also some modifications on the clamp of their microgripper prototypes to reduce adhesive forces. One possible arrangement consists in coating the surface of the grippers with thin layer, such as copper (Cu) or gold (Au), and increasing their roughness, by building particular micropyramids on the endeffector surface.

In the present investigation, the insertion operations of the microgripper jaws into the water drop have been successfully carried out, as the micro-metric tools seemed to be not affected by inflections nor outer-plane instabilities.

Jaws closures and opening have been induced by the comb drives electrostatic actions and the elasticity of the curved beam, respectively. Closure can been regulated by applying to the DUT a voltage form 0 V to 24 V, while opening relies on the elastic energy of deformation that makes the system prone to return to its undeformed configuration. From previous investigations [65], 24 V was confirmed to be the maximum limit for the applied voltage.

Observations during the beads removal from water drops revealed also no problem for keeping the beads in their grasped pose nor for their integrity. It is worth noticing also that phenomena due to water drop surface tension occasionally occurred during drop-to-jaw contact, pointing out the importance of new studies focused on water-DUT interactions which could be helpful to identify all the factors that may influence the evaluation of the real gripping force acting on the agarose beads, such as wet surface adhesion, water gradual evaporation and changes of agarose micro bead size due to water absorbing and retiring.

## 5. Conclusions

This paper provided experimental proofs about the capability of a new micro manipulator to successfully complete three grasping-releasing based tasks in aqueous environment: (i) extraction of a single Agarose micro bead from a drop; (ii) insertion of a single Agarose micro bead into a drop; (iii) grasping of a single Agarose micro bead inside a water drop. These tasks are basic operations that had not yet been tested for the new class of CSFH equipped microgrippers. The results are provided as frames sequences that demonstrate, in an objective and reproducible way, all the above described experimental phases.

## Figures and Tables

**Table 1:** The components of the experimental setup.

Component	Characteristics
DUT	Material: Silicon type P, dopant Boron Orientation <100>, Electrical resistivity: 0.005–0.030 Ohm · cm; Geometry: Overall area: 2000μm×1500μm, Grasping window area: 150μm×150μm, Device thickness: 40 μm, Insulated layer thickness: 3 μm, Handle thickness: 400 μm.
graySuspension	Micro beads: Sepharose CL-4B, Agarose-based, Average diameter (wet state): 45 μm–165 μm. Blue Dye: blue E 132 food coloring solution. Solvent: Deionized water.
grayMicropositioner	n.1 MP25L, range X/Y/Z 10/10/10 mm with 5 μm resolution, n.1 MP25R, range X/Y/Z 10/10/10 mm with 5 μm resolution, n.2 MH-3986-19 X/Y/Z micropositioners with 100 TPI resolution.
grayPower Supply	HP E3631A, DC Output: 0 to +25 V, 0 to −25 V, Resolution: 1.5 mV, Accuracy: 0.04 V at F.S.
grayDUT Stage	Instrumented support with micrometric screws for angular and linear movement of the sample, in the 3 orthogonal directions in space (X, Y, Z).
grayLight Microscope	Eurotek NB50TS NB SOTS, Zoom range: 0.8 ×⋯5× (8×⋯50×), LED illumination Transmitted-Reflected, B2-1525 additional objective 2×.
grayDigital Image	1280 × 720 pixels, 24 bit, 0.988 px/μm.
grayDigital Camera	MD6iS, 6MP, pixel size: 2.8 μm × 2.8 μm, maximum resolution 3264 × 1840 px.

**Table 2:** Operational characteristics of the adopted DUT.

Quantity	Value or Range	Reference	Details
Estimated CSFH Stiffness	0.3 μNm/rad	[52,63]	Theoretical and Numerical approach.
Estimated torque exerted by the comb drive	Up to $10^{-3}\;\mu$Nm	[57]	Theoretical and Numerical approach.
Range: gripper angular displacement	Up to $1.19\pm 0.08^{\circ }$	[64]	measured data from 2 V to 24 V supply voltage.
Resolution: gripper angular displacement	$\geq 0.08^{\circ }$	[64]	measured data from 2 V to 24 V supply voltage. Non-linear response (quadratic curve fitting).
